# Research progress on the effect of traditional Chinese medicine on the activation of PRRs-mediated NF-κB signaling pathway to inhibit influenza pneumonia

**DOI:** 10.3389/fphar.2023.1132388

**Published:** 2023-04-07

**Authors:** Ling Zhang, Xiong Ye, Yuntao Liu, Zhongde Zhang, Xueshan Xia, Shuwei Dong

**Affiliations:** ^1^ The Affiliated Anning First Hospital, Faculty of Life Science and Technology, Kunming University of Science and Technology, Kunming, China; ^2^ The Second Affiliated Hospital of Guangzhou University of Chinese Medicine, Guangdong Provincial Hospital of Chinese Medicine, Guangzhou, China

**Keywords:** TCM, influenza viruses, viral pneumonia, pattern recognition receptor, NF-κB signaling pathway

## Abstract

Influenza pneumonia has challenged public health and social development. One of the hallmarks of severe influenza pneumonia is overproduction of pro-inflammatory cytokines and chemokines, which result from the continuous activation of intracellular signaling pathways, such as the NF-κB pathway, mediated by the interplay between viruses and host pattern recognition receptors (PRRs). It has been reported that traditional Chinese medicines (TCMs) can not only inhibit viral replication and inflammatory responses but also affect the expression of key components of PRRs and NF-κB signaling pathways. However, whether the antiviral and anti-inflammatory roles of TCM are related with its effects on NF-κB signaling pathway activated by PRRs remains unclear. Here, we reviewed the mechanism of PRRs-mediated activation of NF-κB signaling pathway following influenza virus infection and summarized the influence of anti-influenza TCMs on inflammatory responses and the PRRs/NF-κB signaling pathway, so as to provide better understanding of the mode of action of TCMs in the treatment of influenza pneumonia.

## 1 Introduction

Influenza virus is one of the most common respiratory pathogens. According to the estimates of World Health Organization, about 1 billion people worldwide were infected with influenza virus each year, including 3 to 5 million serious cases and 290,000 to 650,000 deaths. The serious cases are often accompanied by viral lung disease, 10% of which even developed viral pneumonia (Cavallazzi and Ramirez, 2018; Riquelme et al., 2022). Previous studies showed that severe pneumonia induced by Influenza virus infection was closely associated with high morbidity and mortality, which posed a significant burden on public health and social development (Gu et al., 2019; Gu et al., 2021; Javanian et al., 2021).

One of the prominent characteristics of severe influenza pneumonia is the incidence of excessive inflammatory responses (Fukuyama and Kawaoka, 2011; Yang et al., 2017). This virus could be recognized by host pattern recognition receptors (PRRs) (Chen et al., 2018; Keilman, 2019), which promotes the activation of transcription factors such as the nuclear factor κB (NF-κB) and downstream production of pro-inflammatory cytokines and chemokines to resist viral infection (Oslund and Baumgarth, 2011; Pinto et al., 2011; Yu et al., 2020). When influenza virus multiplies in large quantities, it may lead to the continuous activation of these signaling pathways, resulting in the overexpression of the inflammatory factors, which would subsequently cause cytokine storm and severe viral pneumonia. More and more studies indicate the essential role of the NF-κB signaling pathway in the virus-induced overproduction of pro-inflammatory factors (Ludwig, 2009; Pandey et al., 2022). How to manipulate the PRR-mediated NF-κB signaling pathway is of great significance for anti-influenza research (Gao et al., 2021).

Traditional Chinese medicine (TCM) has been used for influenza treatment for thousands of years. In TCM theory, influenza was recognized as “plague” (infectious disease) or “current cold,” which was caused by the invasion of external pathogens or environmental changes. Several TCMs, such as Maxing Shigan Decoction, Lianhua Qingwen Capsule, and Jinhua Qinggan Granules, have been recommended by influenza diagnosis and treatment plan (2020 edition) issued by National Health Commission of the People’s Republic of China for clinical influenza treatment to relieve symptoms including chills, fever, cough, headache, and dyspnea (Wu et al., 2020). Compared to chemical drugs, TCMs have advantages including complex ingredients and are tolerant to drug resistance (Wang et al., 2006; Li J. H. et al., 2016; Ding et al., 2017; Zhao et al., 2021a). Further, TCMs have been reported to be involved in regulation of some PRR signaling pathway or inhibiting the nuclear translocation of NF-κB p65 and the expression of pro-inflammatory cytokines and chemokines induced by influenza virus infection. However, the mechanisms of TCMs in treating influenza viral pneumonia *via* regulating the PRR/NF-κB signaling pathway are unclear (Yang et al., 2022). Here, we reviewed the mechanism of influenza-induced TLRs or RIG-I-mediated activation of NF-κB pathway and summarized the effects of TCMs on PRR/NF-κB signaling pathway to provide support for TCM research of anti-influenza and immune regulation.

## 2 The occurrence of influenza pneumonia maybe related to the activation of PRRs-mediated NF-κB signaling pathway

### 2.1 Influenza virus recognition by toll-like receptors (TLRs)

The influenza viral infection could be recognized by different classes of PRRs. The first class of PRRs is toll-like receptors (TLRs). In humans, the family of TLRs consists of 10 members (TLR1-10) and all of them have a N-terminal leucine-rich repeats (LRR) domain which interacts with specific pathogen-associated molecular patterns (PAMPs), a transmembrane domain, and a toll/IL-1 receptor (TIR) domain which is responsible for recruitment of downstream signal adapter proteins (Gao et al., 2013; Kawasaki and Kawai, 2014). In response to ligand binding, TLRs form dimers which recruit particular adaptor proteins for the activation of downstream signaling. All TLRs except TLR3 initiate downstream signaling through the adapter protein myeloid differentiation primary response 88 gene (MyD88)-dependent pathway. For TLR4, it uses TIR domain containing adaptor protein (TIRAP) as additional adapter in this pathway. The activation of MyD88 leads to the phosphorylation of IL-1R-associated kinases 1 and 4 (IRAK1/4) which binds with TNF receptor-associated factor 6 (TRAF6) to mediate the activation of the transforming growth factor β-activated kinase 1 (TAK1)/TAK1-binding protein (TAB) complex, and then induces the translocation of transcription factors such as NF-κB to regulate the expression of inflammatory cytokine genes (Kawai and Akira, 2007; Jiang et al., 2020). Besides MyD88-dependent pathway, TLR3 recruits TIR-domain-containing adapter-inducing interferon-β (TRIF) and the endocytosed TLR4 recruits TRIF and TRIF-related adaptor molecule (TRAM), which activates TRIF-dependent pathway. The interaction between TRIF and TRAF6 recruits the receptor-interacting protein 1 (RIP1) and activates the TAK1/TAB complex that leads to the activation of NF-κB and downstream inflammatory responses. Alternatively, TRIF interacts with TRAF3 and recruits TANK (TRAF family member associated NF-κB activator)-binding kinase 1 (TBK1) and the inhibitor of NF-κB (IκB) kinase ε (IKKε), which drives the phosphorylation of the interferon regulatory factor 3 (IRF3) or IRF7 and downstream production of type I interferons (IFNs) (Kawai and Akira, 2007; Owen et al., 2020).

TLR3, TLR4, TLR7, and TLR8 have been reported to play a role in influenza virus infection. Continuous activation of TLR3 has been found both in patients that died in the 2009 pandemic and in the lungs of C57BL/6 mice infected with influenza virus, while the deletion of TLR3 in mice was associated with reduced chemokine expression and longer survival period (Le Goffic et al., 2006; Mauad et al., 2010). TLR4/MyD88 signaling was suggested to affect influenza virus infection because reduction of viral titers was detected in cells treated with an anti-TLR4 antibody or in MyD88 knockout cells (Marchant et al., 2010). Mice with TLR4 deficiency had increased survival from lethal infection of influenza virus and the administration of TLR4 antagonist eritoran reduced viral titers and cytokine expression and improved pulmonary pathological changes and pulmonary function (Shirey et al., 2013). The use of a TLR7/8 agonist inhibited influenza virus replication and induced IFN production both *in vitro* and *in vivo* (Hammerbeck et al., 2007). TLR7 recognizes ssRNA derived from influenza virus, whereas the relevance of TLR8 in this viral infection still needs more investigation (Iwasaki and Pillai, 2014).

### 2.2 Influenza virus recognition by RIG-I

Retinoic acid-inducible gene I (RIG-I) is another class of PRR detecting viruses within the cytosol of infected cells. It contains a pair of N-terminal caspase activation and recruitment domains (CARDs), the Helicase domain in which there are two RecA domains and an alpha-helical insertion domain (Hel2i), and a C-terminal domain. In resting cells, RIG-I locks in an autorepressed conformation by clamping the CARDs to the surface of Hel-2 (Thoresen et al., 2021). On detection of viral RNA, the Helicase domain of RIG-I binds to ATP, which promotes conformational changes allowing the CARDs to connect to mitochondrial antiviral signaling proteins (MAVS). MAVS then assembles with TRAF2/3/6, TBK1, and IKK complex to function as a signaling hub which drives the activation of IRF3, IRF7, and NF-κB and downstream production of IFNs and proinflammatory cytokines and chemokines (Iwasaki and Pillai, 2014; Thoresen et al., 2021).

In cells infected with influenza virus, RIG-I mainly recognizes viral nucleocapsids and viral genomic RNA bearing 5′-ppp (Rehwinkel et al., 2010). RIG-I initiates several anti-influenza defense mechanisms which include the production of type I IFNs, direct inhibition of virus replication, and promoting the assembly of an inflammasome complex to aid in viral clearance (Weber-Gerlach and Weber, 2016). It has been reported that the suppression of RIG-I pathway is common in children with severe influenza virus infection and is associated with increased risk of clinical complications (Novak et al., 2020). In addition, influenza virus has been suggested to disrupt RIG-I dependent signaling through MAVS that several subunits of RNA polymerase of this virus have an ability to translocate to the mitochondria and impair innate immunity (Weis and Te Velthuis, 2021).

### 2.3 The activation of NF-κB pathway and overproduction of pro-inflammatory factors following influenza virus infection

The NF-κB pathway has long been recognized as a prototypical proinflammatory signaling pathway. NF-κB is an evolutionarily conserved transcription factor. In mammals, there are five members in the NF-κB family, namely p50, p52, p65 (RelA), RelB, and c-Rel, of which p65 and p50 form a heterodimer and largely involve in the classical pathway of NF-κB activation. In the resting state, NF-κB p65:p50 dimers are sequestered in the cytoplasm in an inactive state by members of the IκB proteins. Once engaged with an activating signal, the IκB proteins are phosphorylated by IKK complex and this leads to ubiquitination and subsequent proteasomal degradation of the IκB, and then the NF-κB dimer is released and translocates into the nucleus so that to promote the transcription of pro-inflammatory genes such as TNF-α, IL-6, IL-8, IFN-β, and IL-1β (Mitchell et al., 2016; Mitchell and Carmody, 2018).

**FIGURE 1:**
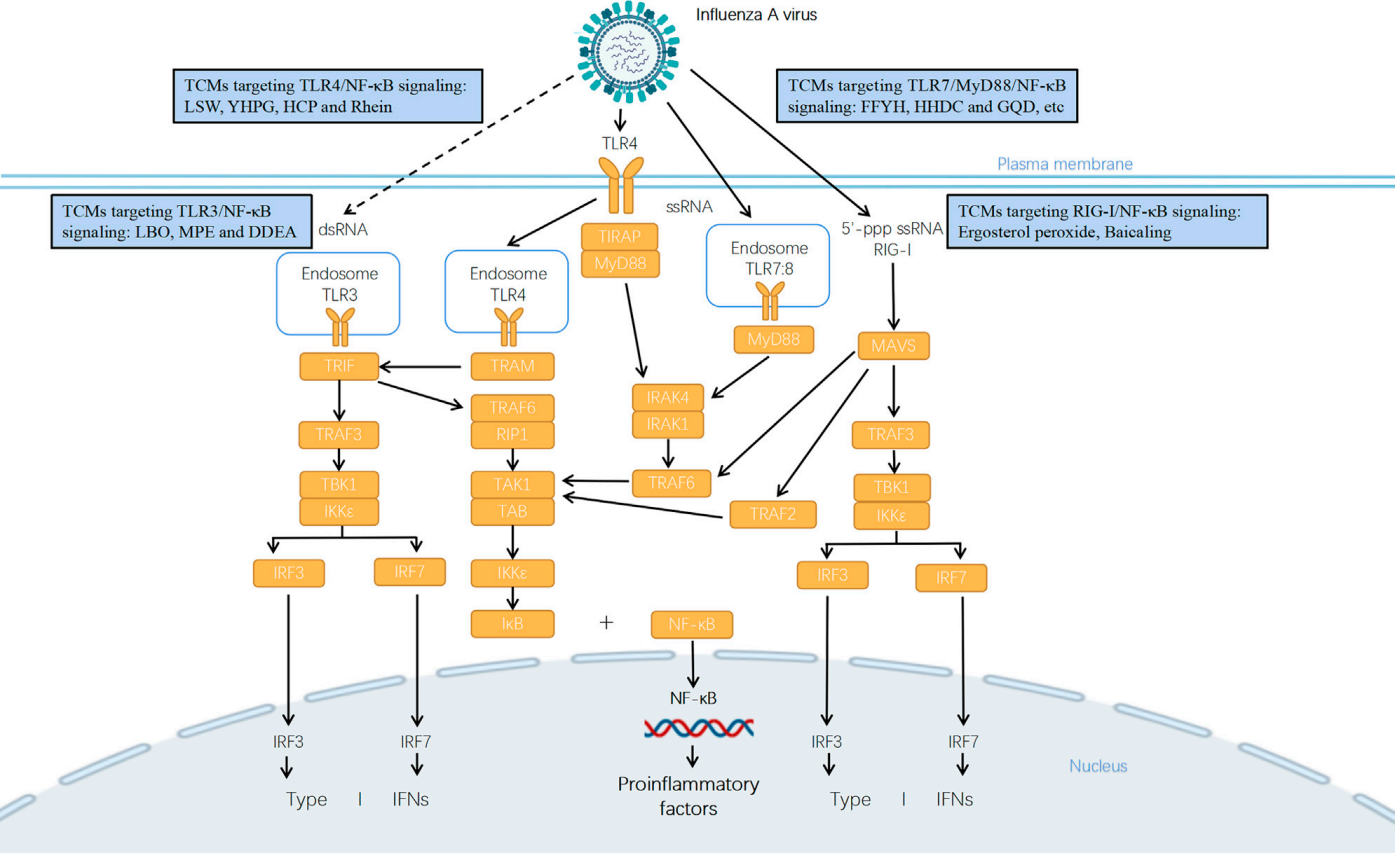
Potential mechanisms of anti-influenza TCMs in treatment of influenza pneumonia *via* TLRs/RIG-I signaling pathway. Influenza virus infection is detected by multiple PRRs such as TLRs and RIG-I. TLR3 recruits TIR-domain-containing adapter-inducing interferon-β (TRIF) and the endocytosed TLR4 recruits TRIF and TRIF-related adaptor molecule (TRAM), which activates TRIF-dependent pathway. The interaction between TRIF and TNF receptor-associated factor 6 (TRAF6) recruits the receptor-interacting protein 1 (RIP1) and activates the transforming growth factor β-activated kinase 1 (TAK1)/TAK1-binding protein (TAB) complex that leads to activation of NF-κB and downstream transcription of pro-inflammatory genes. Alternatively, TRIF interacts with TRAF3 and recruits TANK (TRAF family member associated NF-κB activator)-binding kinase 1 (TBK1) and the inhibitor of NF-κB (IκB) kinase ε (IKKε), which drives the phosphorylation of the interferon regulatory factor 3 (IRF3) or IRF7 and downstream production of type I interferons (IFNs). TLR3 recognizes dsRNA in endosomes, but it recognizes currently unknown RNA structures presenting in influenza virus-infected cells (presented in dotted line). TLR4, TLR7, and TLR8 initiate downstream signaling through the adapter protein myeloid differentiation primary response differentiation gene 88 (MyD88)-dependent pathway. For TLR4, it uses TIR domain containing adaptor protein (TIRAP) as additional adapter in this pathway. TLR7 recognizes the ssRNA genomes of influenza virus taken up into the endosome. The activation of MyD88 leads to the phosphorylation of IL-1R-associated kinases 1 and 4 (IRAK1/4) which binds with TRAF6 to mediate the activation of TAK1/TAB complex, and then induces the translocation of NF-κB to regulate the expression of cytokines and chemokines. RIG-I mainly recognizes viral nucleocapsids and viral genomic RNA bearing 5′-ppp. It connects to MAVS which then assembles with TRAF2/3/6, TBK1, and IKK complex to function as a signaling hub that drives the activation of IRF3, IRF7, and NF-κB and downstream production of IFNs and proinflammatory cytokines and chemokines. A variety of TCMs could regulate the expression of key components in TLRs/RIG-I signaling pathways so as to inhibit viral replication and virus-induced pro-inflammatory response.

The NF-κB signaling pathway is continuously activated when influenza viruses multiply in large quantities, which leads to excess expression of pro-inflammatory genes (Wurzer et al., 2004; Vitiello et al., 2012; Ramos and Fernandez-Sesma, 2015; Pandey et al., 2022) (Figure 1). In patients with influenza pneumonia including 2009 H1N1, H1N1, H3N1, and H7N1, higher levels of proinflammatory cytokines and chemokines were detected, such as IL-6, IL-8, and TNFs (To et al., 2010; Xie et al., 2021). Overproduction of proinflammatory cytokines and chemokines induced by influenza virus infection has been suggested to relate to widespread tissue damage (Liu et al., 2016; Karki and Kanneganti, 2021). Based on the fact that the majority of immunoreceptors including TLRs and RIG-I control the expression of diverse inflammatory genes *via* the activation of NF-κB, it is highly possible that PRRs-mediated NF-κB activation plays a pathogenic role in influenza pneumonia.

## 3 The effects of TCMs on PRRs-mediated activation of NF-κB induced by influenza virus infection

The composition, effective dose, cell and animal models, virus strain, drug used as a positive control, antiviral and anti-inflammatory effects, and possible mechanism of TCMs and their ingredients against influenza virus infection and influenza pneumonia have been summarized in Figure 1 and Tables 1–3.

**TABLE 1:** The effects of TCM formulae on PRRs-mediated activation of NF-κB induced by influenza virus infection.

TCM formula	Effective dose	Cell/Animal	Virus	Drug used as a positive control	Antiviral effect	Anti-inflammatory effect	Molecule target	References
FFYH	177.8–354.8 μg/mL	A549 MDCK	FM1, PR8, WSN and H5N1/SY/2004	LH	Inhibited H1N1 and H5N1 induced CPE in MDCK.		TLR7, MyD88, IRF7 and TRAF6	Zhang Y. et al. (2021)
				RBV				
				OSE				
	1.0, 0.5 g/kg	ICR mice	FM1	LH	Increased the death protection rate	TNF-α, IL-6, IFN-α, IL-1β and IP-10	TLR7, MyD88, IRF7, IRAK4, TRAF3 and TRAF6	
				RBV				
HHDC	16, 8 or 4 g/kg	ICR mice	PR8	OSE	Alleviated the weight loss and reduced the virus titers	IL-6, IFN-γ, and TNF-α	TLR7, TLR3, MyD88, NF-κB p65 and TRAF3	Zhang L. et al. (2021), Zhang et al. (2022)
						Reduced lung index and improved lung histopathology		
LSW	1.192 μg/mL	A549	PR8	OSE	Inhibited virus proliferation in MDCK.	IL-1β, IL-6, TNF-α and IFN-γ	TLR4, phospho-NF-κB p65, and phospho-IκBα	Ma et al. (2020), Zhao et al. (2021a)
	100, 50 mg/kg	Balb/c mice	PR8	OSE	Improved the survival rate and alleviated virus-induced lung lesions. Decreased the virus titer	IL-1β, IL-6, TNF-α and IFN-γ		
						Reduced inflammatory cell infiltration in lung		
GQD	0.19 g/d	C57BL/6 mice TLR7^−/−^ mice	FM1	OSE	Improved the survival rate and decreased the virus titer in lungs	Reduce lung index and inflammatory cell infiltration	TLR7, MyD88, IRAK4, TRAF6, and NF-κB p65	Shi et al. (2020), Deng et al. (2021)
XJXRY	92 mg/d	C57BL/6 mice TLR7^−/−^ mice	FM1	OSE	Alleviated the weight loss	Reduced lung index	TLR7, MyD88, and NF-κB p65	Li et al. (2018), Fu et al. (2019)
Guizhi-and-Mahuang decoction	140 mg/d	C57BL/6 mice TLR7^−/−^ mice	FM1	OSE	Alleviated the weight loss	Reduce lung index and inflammatory cell infiltration	TLR7, MyD88, IRAK4 and NF-κB p65	Fu et al. (2018)
Yin-Qiao San	224 mg/d	C57BL/6 mice TLR7^−/−^ mice	FM1	OSE	Alleviated the weight loss	Reduced lung index and inflammatory cell infiltration	TLR7, MyD88, IRAK4 and NF-κB p65	Fu et al. (2018)
YHPG	7.5, 15, 30 g/kg	ICR mice	PR8	RBV	Reduced pulmonary viral load	IL-4, IL-5 and TNF-α	TLR4, MyD88, TRAF6 and NF-κB p65	Peng et al. (2016a), Peng et al. (2016b)
LBO	25–100 μg/mL	MDCK	WSN	RBV	Inhibited viral proliferation	IL-1β, IL-6, and IFN-β	Phospho-NF-κB p65 and phospho-IRF3	Mao et al. (2021)
		A549	FM1					
	100 μg/mL	Balb/c mice	FM1	RBV		IL-1β and IL-6		
						Alleviated inflammatory cell infiltration in lung, and reduced lung index		
KBD	13 mg/kg and 26 mg/kg	Restraint stress + Kunming mice	FM1	OSE	Increased the death protection rate	IFN-β	MAVS, phospho-IRF3 and NF-κB p65	Chen et al. (2017)
						Alleviated inflammatory cell infiltration in lung and reduced the lung index		

Abbreviations: fufang yinhua jiedu (FFYH), Madin-Darby canine kidney (MDCK), influenza virus A/FortMonmouth/1/47 (H1N1) (FM1), influenza virus A/WSN/1933 (H1N1) (WSN), influenza virus A/Puerto Rico/8/34 (H1N1) (PR8), influenza virus A/Duck/Jiangsu/Sheyang/2004 (H5N1) (H5N1/SY/2004), Lianhua Qingwen (LH), ribavirin (RBV), oseltamivir (OSE), cytopathic effect (CPE), toll-like receptors (TLRs), myeloid differentiation primary response differentiation gene 88 (MyD88), the interferon regulatory factors (IRFs), tumornecrosis factors-α (TNF-α), TNF receptor-associated factors (TRAFs), Interleukins (ILs), interferons (IFNs), interferon-gamma (IFN-gamma)-induced protein 10 (IP-10), IL-1R-associated kinases (IRAKs), Huanglian-Houpu drug combination (HHDC), the nuclear factor κB (NF-κB), Liu Shen Wan (LSW), the inhibitor of NF-κB (IκB) α (IκBα), Gegen Qin lian decoction (GQD), Xinjiaxiangruyin (XJXRY), Yinhuapinggan granule (YHPG), Luofushan-Baicao Oil (LBO), KangBingDu oral liquid (KBD), mitochondrial antiviral signaling proteins (MAVS).

**TABLE 2:** The effects of single-active-ingredients of TCM on PRRs-mediated activation of NF-κB induced by influenza virus infection.

The single-active-ingredients of TCM	Effective dose	Cell/Animal	Virus	Positive drug	Antiviral effect	Anti-inflammatory effect	Molecule target	References
Carvacrol	50 mg/kg/d	C57BL/6 mice	FM1	RBV	Alleviated weight loss and decreased viral load in lung	IFN-γ, IL-1, IL-2, IL-4, IL-5, IL-6, IL-10, IL-12 and TNF-ɑ	TLR7, IRAK4, TRAF6, MAVS and IRF3	Zheng et al. (2021)
						Improved lung histopathological changes and decreased lung index		
C8	19.9–91.4 μg/mL	A549	PR8		Inhibited viral proliferation in MDCK	IL-6, IL-8, TNF-α, IP-10, MCP-1 and CCL5	TLR7, MyD88, TRAF6, NF-κB p65	Guan et al. (2017), Wang et al. (2017), Wang Y. et al. (2018)
			H6N2/GD/2009					
			H7N3/GD/1994					
			H9N2/GD/1996					
Berberine	≥20 μg/mL	A549	FM1	RBV	Inhibited the virus titer in A549 and FM1 induced CPE in MDCK			Wu Y. et al. (2011), Yan et al. (2018)
	20 mg/kg/d	C57BL/6 mice	FM1	OSE	Inhibited weight loss and viral replication	MCP-1, IFN-γ and TNF-α	TLR7, MyD88, and NF-κB p65	
						Relieved pathological changes		
Forsythoside A	20 mg/kg/d	C57BL/6 and TLR7^−/−^ mice	FM1	OSE	Reduced the virus titer. Alleviated weight loss	Reduced lung index and inflammatory cell infiltration in lung	TLR7, MyD88, TRAF6, IRAK4 and NF-κB p65	Deng et al. (2016), Zheng et al. (2022)
HCP	20, 40 mg/kg/d	BALB/c mice	FM1	RBV	Improve the survival rate of infected mice	TNF-α, IL-6, IFN-α, CCL-5, MCP-1, MIP-1α and IP-10	TLR4 and NF-κB p65	Zhu et al. (2018), Ling et al. (2023)
						Alleviated acute lung injury		
Ergosterol peroxide	100–300 μg/mL	A549	PR8			TNF-α, IL-6, IL-8, IP-10, CCL5, MCP-1, and MIP-1α	RIG-I and NF-κB p65	Zhou et al. (2019)
		293T						
Baicalin	20–80 μg/mL	MDCK	FM1 H3N2/BJ/32/92	RBV	Inhibited viral proliferation	Inhibited neuraminidase activity		Ding et al. (2014), Pang et al. (2018)
				OSE				
	15 mg/kg	C57BL/6 mice	FM1	RBV	Alleviated weight loss	IFN-γ, IL-1β, IL-2, IL-5, and IL-6	RIG-I, IRF3, IRF7 and NF-κB	
						Reduced the lung index and alleviated pulmonary damage		
Rhein	2.5–10 μg/mL	A549	PR8	RBV	Inhibited viral proliferation	IL-1β, IL-6, IL-8 and TNF-α	TLR2, TLR3, TLR4 and NF-κB p65	Wang Q. W. et al. (2018)
		MDCK	ST169					
	75 or 150 mg/kg/d	C57BL/6 mice	PR8	OSE	Improved the survival rate	IL-1β, IL-6, IL-8, TNF-α		
						Reduced the lung index and alleviated lung histopathological changes		
MPE	10 or 20 mg/kg	ICR mice	PR8	RBV	Reduced the virus titer	Reduced the lung index	TLR3 and NF-κB p65	Wu X. N. et al. (2011)
Patchouli alcohol	10 μg/mL	HBE	FM1	Zanamivir	Inhibited viral proliferation	IFN-γ and IL-4	RIG-1, MAVS, IRF3 and IRF7	Kiyohara et al. (2012), Wu et al. (2013)
DDEA	IC_50_ > 100 μM	A549	PR8		Unable to inhibit viral replication	TNF-α, IFN-α, IFN-β, IL-6, MCP-1, IL-8, IP-10, MIP-1α and MIP-1β	TLR-3, RIG-I, IRF3 and NF-κB p65	Liu S. J. et al. (2015), Li et al. (2021)
		U937						

Abbreviations: influenza virus A/FortMonmouth/1/47 (H1N1) (FM1), ribavirin (RBV), interferons (IFNs), Interleukins (ILs), tumornecrosis factors-α (TNF-α), toll-like receptors (TLRs), IL-1R-associated kinases (IRAKs), TNF receptor-associated factors (TRAFs), mitochondrial antiviral signaling proteins (MAVS), the interferon regulatory factors (IRFs), influenza virus A/Puerto Rico/8/34 (H1N1) (PR8), influenza virus A/Duck/Guangdong/2009 (H6N2) (H6N2 /GD/2009), influenza virus A/Duck/Guangdong/1994 (H7N3) (H7N3/GD/1994), influenza virus A/Chicken/Guangdong/1996 (H9N2) (H9N2/GD/1996), Madin-Darby canine kidney (MDCK), interferon-gamma (IFN-gamma)-induced protein 10 (IP-10), monocyte chemoattractant protein-1(MCP-1), chemokine ligand 5 (CCL-5), myeloid differentiation primary response differentiation gene 88 (MyD88), the nuclear factor κB (NF-κB), oseltamivir (OSE), macrophage inflammatory protein (MIP)-1α/1β (MIP-1α/1β), retinoic acid-inducible gene I (RIG-I), influenza virus A/Beijing/32/92 (H3N2) (H3N2/BJ/32/92), influenza virus A/Shantou/169/06 (H1N1) (ST169), human respiratory epithelial cell (HBE), 50% inhibitory concentration (IC_50_), U937-derived macrophages (U937).

**TABLE 3:** The composition of formulae and single-active-ingredients of TCM.

TCM	Botanical drugs (Latin name)	Botanical drugs (Chinese Pinyin)	Botanical drugs (English name)	Family	References
FFYH	*Artemisia annua* L.	Qinghao	Artemisiae Annuae Herba	Asteraceae	Zhang Y. et al. (2021)
	*Lonicerae japonica* Thunb.	Jinyinhua	Lonicerae Flos	Caprifoliaceae	
	*Nepeta tenuifolia* Benth.	Jingjie	Schizonepetae Herba	Lamiaceae	
	*Mentha canadensis* L.	Bohe	Menthae Haplocalycis Herba	Lamiaceae	
	*Chrysanthemum indicum* L.	Juhua	Chrysanthemi Indici Flos	Asteraceae	
	*Isatis tinctoria* L.	Daqingye	Isatidis Folium	Brassicaceae	
	*Forsythia suspensa* (Thunb).Vahl	Lianqiao	Forsythiae Fructus	Oleaceae	
	*Commelina communis* L.	Yazhicao	Commelinae Herba	Commelinaceae	
	*Glycine max* (L.) Merr.	Dandouchi	Sojae Semen Praeparatum	Fabaceae	
	*Peucedanum praeruptorum* Dunn	Qianhu	Peucedani Radix	Apiaceae	
HHDC	*Coptis chinensis* Franch	Huanglian	Coptidis Rhizoma	Ranunculaceae	Zhang L. et al. (2021)
	*Magnolia officinalis*	Houpu	Officinalis Cortex	Magnoliaceae	
LSW	the gall-stone of *Bos taurus* domesticus Gmelin	Niuhuang	Calculus Bovis	Bovine	Ma et al. (2007), Ma et al. (2020)
	the excretion of *Moschus*	Shexiang	Musk	Moschidae	
	the excretion of *Venenum Bufonis*	Chansu	Cinobufagin Venom Toad	Bufonidae	
	the shell of *Pernulo*	Zhenzhu	Pearl		
	N/A	Xionghuang	Realgar		
	N/A	Longnao	Borneol		
GQD	*Pueraria lobata* (Willd.) Ohwi	Gegen	Radix Puerariae	Fabaceae	Shi et al. (2020),
	*Scutellaria baicalensis* Georgi	Huangqin	Radix Scutellariae	Lamiaceae	
	*C. chinensis* Franch	Huanglian	Coptidis Rhizoma	Ranunculaceae	
	*Glycyrrhiza glabra* L.	Gancao	Radix Glycyrrhizae	Fabaceae	
XJXRY	*Mosla chinensis* Maxim	Shixiangru	Herba Moslae	Lamiaceae	Fu et al. (2019)
	L. *japonica* Thunb	Jinyinhua	Flos Lonicerae	Caprifoliaceae	
	*Lablab purpureus*	Biandou	Dolichos	Fabaceae	
	*M. officinalis*	Houpu	Officinalis Cortex	Magnoliaceae	
	*F. suspensa* (Thunb.) Vahl	Lianqiao	Fructus Forsythiae	Oleaceae	
Guizhi-and-Mahuang decoction	*Ephedra sinica* Stapf	Mahuang	Herba Ephedrae	Ephedraceae	Fu et al. (2018)
	*Neolitsea cassia*	Guizhi	Ramulus Cinnamomi	Lauraceae	
	*Prunus armeniaca*	Kuxingren	Semen Armeniacae Amarum	Rosaceae	
	*G. glabra* L.	Gancao	Radix Glycyrrhizae	Fabaceae	
	*G. glabra* L.	Zhigancao	Radix Glycyrrhizae Preparata	Fabaceae	
	*Paeonia lactiflora* Pall	Baishao	Radix Paeoniae Alba	Paeoniaceae	
	*Zingiber officinale* Roscoe	Shengjiang	Rhizoma Zingiberis Recens	Zingiberaceae	
	*Ziziphus jujuba* Mill	Dazao	Jujube	Rhamnaceae	
Yin-Qiao San	*F. suspensa* (Thunb.) Vahl	Lianqiao	Fructus Forsythiae	Oleaceae	Fu et al. (2018)
	*L. japonica* Thunb	Jinyinhua	Flos Lonicerae	Caprifoliaceae	
	*Platycodon grandiflorus*	Jigeng	Radix Platycodonis	Campanulaceae	
	*M. canadensis* L.	Bohe	Herba Menthae	Lamiaceae	
	*Lophatherum gracile* Brongn	Danzhuye	Herba Lophatheri	Poaceae	
	*G. glabra* L.	Gancao	Radix Glycyrrhizae	Fabaceae	
	*N. tenuifolia* Benth	Jingjiesui	Herba Schizonepetae	Lamiaceae	
	*G. max* (L.) Merr.	Dandouchi	Fermented soybean	Fabaceae	
	*Arctium lappa* L.	Niubangzi	Fructus Arctii	Asteraceae	
	*Phragmites australis*	Lugen	Rhizoma Phragmitis	Poaceae	
YHPG	*P. lobata* (Willd.) Ohwi	Gegen	Puerariae Lobatae Radix	Fabaceae	Peng et al. (2016a), Peng et al. (2016b)
	*L. japonica* Thunb	Jinyinhua	Flos Lonicerae Japonicae	Caprifoliaceae	
	*Reynoutria japonica* Houtt	Huzhang	Polygoni Cuspidati Rhizoma	Polygonaceae	
	*E. sinica* Stapf	Mahuang	Herba Ephedrae	Ephedraceae	
	*P. armeniaca*	Kuxingren	Semen Armeniacae Amarum	Rosaceae	
	*G. glabra* L	Gancao	Radix Glycyrrhizae	Fabaceae	
KBD	*I. tinctoria L*.	Banlangen	Isatidis Radix	Brassicaceae	Chen et al. (2017)
	*P. australis*	Lugen	Rhizoma Phragmitis	Poaceae	
	*Rehmannia glutinosa* (Gaertn.) DC.	Dihuang	Radix Rehmanniae	Orobanchaceae	
	*Curcuma aromatica* Salisb	Yujin	Radix Curcumae	Zingiberaceae	
	*Anemarrhena asphodeloides* Bunge	Zhimu	Rhizoma Anemarrhenae	Asparagaceae	
	*Acorus calamus*	Shichangpu	Rhizoma Acori Tatarinowii	Acoraceae	
	*Pogostemon cablin* (Blanco) Benth	Guanghuoxiang	Herba Pogostemonis	Lamiaceae	
	*F. suspensa* (Thunb.) Vahl	Lianqiao	Fructus Forsythiae	Oleaceae	
	N/A	Shigao	Gypsum Fibrosum	N/A	
Carvacrol	*M. chinensis* Maxim	Shixiangru	Herba Moslae	Lamiaceae	Zheng et al. (2021)
C8	*Laggera crispata*	Choulingdan	Laggerae Herba	Asteraceae	Wang Y. et al. (2018)
Berberine	*C. chinensis* Franch	Huanglian	Coptidis Rhizoma	Ranunculaceae	Wu Y. et al. (2011)
Forsythoside A	*F. suspensa* (Thunb.) Vahl	Lianqiao	Forsythiae Fructus	Oleaceae	Zheng et al. (2022)
HCP	*Houttuynia cordata* Thunb	Yuxingcao	Houttuyniae Herba	Saururaceae	Zhu et al. (2018)
Ergosterol peroxide	*Strobilanthes cusia* (Nees) Kuntze	Nanbanlangen	Baphicacanthis Cusiae Rhizoma et Radix	Acanthaceae	Zhou et al. (2019)
Baicalin	*S. baicalensis* Georgi	Huangqin	Radix Scutellariae	Lamiaceae	Pang et al. (2018)
Rhein	*Rheum palmatum* L.	Dahuang	Rhei Radix et Rhizoma	Polygonaceae	Wang Q. W. et al. (2018)
MPE	*M. officinalis*	Houpu	Officinalis Cortex	Magnoliaceae	Wu X. N. et al. (2011)
Patchouli Alcohol	*P. cablin* (Blanco) Benth	Guanghuoxiang	Herba Pogostemonis	Lamiaceae	Wu et al. (2013)
DDEA	*Ophiocordyceps sinensis*	Dongchongxiacao	Cordyceps	Ophiocordycipitaceae	Li et al. (2021)

Abbreviations: fufang yinhua jiedu (FFYH), Huanglian-Houpu drug combination (Magnoliae officinalis cortex) (HHDC), Liu Shen Wan (LSW), Gegen Qinlian decoction (GQD), Xinjiaxiangruyin (XJXRY), Yinhuapinggan granule (YHPG), KangBingDu oral liquid (KBD), polysaccharides isolated from Houttuynia cordata (HCP), polyphenol extract from the bark of Magnolia officinalis (MPE), (2Z,4E)-deca-2,4-dienoic acid (DDEA).

### 3.1 Traditional Chinese medicine formulas

#### 3.1.1 Fufang yinhua jiedu (FFYH) granules

FFYH is a Chinese patent medicine optimized based on Yin-Qiao-San. It consists of *Artemisia annua* L., *Lonicerae japonica* Thunb., *Nepeta tenuifolia* Benth., *Mentha canadensis* L., *Chrysanthemum indicum* L., *Isatis tinctoria* L., *Forsythia suspensa* (Thunb.) Vahl., *Commelina communis* L., *Glycine max* (L.) Merr., and *Peucedanum praeruptorum* Dunn. FFYH exhibits antiviral activity against several influenza A viruses, such as H1N1, H3N2, H5N1, H7N9, and H9N2, through cytopathic effect inhibition assay and hemagglutination tests *in vitro*. ICR mice infected with influenza virus A/FortMonmouth/1/47 (H1N1) (FM1) showed wrinkled hair, reduced feed intake and weight loss, respiratory distress, and the serum levels of TNF-α, IL-6, IP-10, IFN-γ, and IL-1β were significantly elevated. FFYH treatment alleviated the above disease symptoms and acute lung injury, and significantly reduced the expression levels of serum TNF-α and other inflammatory factors in infected mice. Moreover, the upregulation of expression of TLR7, MyD88, IRF7, IRAK4, TRAF6, and TRAF3 induced by viral infection were significantly suppressed under FFYH treatment both *in vitro* and *in vivo*. The inhibition of ‘cytokine storm’ *via* the regulation of TLR7/MyD88 signaling pathway was suggested as a potential mechanism of FFYH against influenza pneumonia (Zhang Y. et al., 2021).

#### 3.1.2 Huanglian-Houpu drug combination (HHDC)

HHDC is a botanical drug pair containing huanglian (*Coptis chinensis* Franch.) and houpu (the bark of *Magnolia officinalis*), which has a good effect on dysentery and gastrointestinal diseases (Luo et al., 2019; Wang et al., 2019; Zhang et al., 2022). The active ingredients in HHDC are berberine hydrochloride and magnolol *via* high-performance liquid chromatography (HPLC) analysis and these two components were suggested as the main anti-influenza components in HHDC. HHDC significantly reduced the viral load, pulmonary virus titer, and the level of inflammatory cell infiltration in the lung tissues of mice infected with influenza virus A/Puerto Rico/8/34 (H1N1) (PR8), as well as significantly reduced the serum levels of IL-6, IFN-γ, and TNF-α in a dose-dependent manner. The mRNA and protein expression levels of TLR7, TLR3, MyD88, NF-κB p65, and TRAF3 in the lungs of mice received high-dose HHDC treatment were significantly lower than those of the virus-infected group (Zhang L. et al., 2021). Therefore, HHDC may act against influenza pneumonia by regulating the TLR/MyD88/NF-κB signaling pathway.

#### 3.1.3 Liu Shen Wan (LSW)

LSW is a classic Chinese patent medicine having anti-inflammatory and analgesic activities (Ma et al., 2007). The main ingredients of LSW are the gall-stone of *Bos taurus* domesticus Gmelin, the excretion of *Moschus*, the excretion of *Venenum Bufonis*, the shell of *Pernulo*, realgar, and borneol. It has been widely used for over a century to treat influenza, tonsillitis, and pharyngitis (Zhao et al., 2021b). In MDCK and A549 cells infected with PR8, LSW inhibited viral proliferation as well as significantly downregulated the expression levels of IL-1β, TNF-α, IL-6, and IFN-γ. In mice infected with PR8, LSW significantly improved the survival rate and reduced viral titers and the secretion of TNF-α, IL-1β, IL-6, and IFN-γ in pulmonary tissue. Moreover, LSW significantly reduced the expression level of TLR4, phospho-NF-κB p65, and phospho-IκBα but upregulated IκBα expression both *in vivo* and *in vitro* (Ma et al., 2020). It is suggested that LSW exhibited the antiviral and anti-inflammatory effects by regulating the TLR4/NF-κB signaling pathway.

#### 3.1.4 Gegen Qinlian decoction (GQD)

GQD is composed of the root of *Pueraria loba*ta (Willd.) Ohwi, *Scutellaria baicalensis* Georgi, *C. chinensis* Franch., and *Glycyrrhiza glabra* L. (Zhang et al., 2016; Deng et al., 2021). It has anti-bacterial and anti-inflammatory effects and is widely used in the treatment of fever and cough (Yu et al., 2005; Kwon et al., 2008; Nayak et al., 2014; Zhang et al., 2016; Deng et al., 2021). GQD can inhibit the proliferation of the FM1 strain, improve the survival rate, and significantly reduce the virus-induced mRNA expression levels of TLR7, MyD88, IRAK4, TRAF6, and NF-κB p65 in wild-type C57BL/6 mice. However, in TLR7-deficient mice, the treatment of GQD has no effect on the upregulated expression levels of MyD88 and NF-κB p65 induced by the virus infection. GQD has been proposed playing antiviral and immunomodulatory roles against influenza pneumonia by affecting the TLR7 signaling pathway (Shi et al., 2020).

#### 3.1.5 Xinjiaxiangruyin (XJXRY)

XJXRY is often used in the clinical treatment of influenza in summer (Li et al., 2018). It consists of *Mosla chinensis* Maxim, *L. japonica* Thunb, *Lablab purpureus*, the bark of *M. officinalis*, and *F. suspensa* (Thunb.) Vahl. It effectively reduced lung inflammation and downregulated the mRNA and protein expression levels of TLR7, MyD88, and NF-κB p65 in C57BL/6 mice infected with FM1 in a hot and humid environment (Fu et al., 2019). It was suggested that XJXRY may reduce virus-induced lung injury by downregulating the expression levels of key factors in TLR7 signaling pathway.

#### 3.1.6 Guizhi-and-Mahuang decoction

Guizhi-and-Mahuang decoction has anti-influenza, antipyretic, analgesic, anti-inflammatory, and anti-asthma activities (Du et al., 2013). It consists of *Ephedra sinica* Stapf, *Neolitsea cassia*, *Prunus armeniaca*, *G. glabra* L., *Paeonia lactiflora* Pall., *Zingiber officinale* Roscoe, and *Ziziphus jujuba* Mill. It was found that this decoction had a protective effect on FM1-infected mice by reducing the lung index and viral loads, as well as inhibiting the expression levels of TLR7, MyD88, IRAK4, and NF-κB p65. In TLR7-deficient mice, viral infection still induced the mRNA expression level of MyD88 and NF-κB but they were lower than those in wild type mice. Moreover, the mRNA expression levels of these genes were similar between viral infection group and Guizhi-and-Mahuang decoction treatment group (Fu et al., 2018). The regulation of TLR7/NF-κB signaling pathway by Guizhi-and-Mahuang decoction is likely to play an important role in the treatment of influenza-induced viral pneumonia.

#### 3.1.7 Yin-Qiao San

Yin-Qiao San is a classic TCM with antipyretic and anti-inflammatory functions and used for prevention and control of viral infectious diseases (Zhang et al., 2019). It consists of *F. suspensa* (Thunb.) Vahl, *L. japonica* Thunb., *Platycodon grandiflorus*, *M. canadensis* L., *Lophatherum gracile* Brongn., *G. glabra* L., *N. tenuifolia* Benth., *G. max* (L.) Merr., *Arctium* lappa L., and *Phragmites australis*. In wild type mice, FM1 infection significantly upregulated the levels of TLR7, MyD88, IRAK4 and NF-κB while the treatment of Yin-Qiao San statistically reduced virus-induced expression of these genes. In TLR7-deficient mice, Yin-Qiao San failed to inhibit upregulation of NF-κB under viral infection. Yin-Qiao San may target some elements in the TLR7/MyD88/NF-κB pathway to achieve its therapeutic effects against influenza pneumonia (Fu et al., 2018).

#### 3.1.8 Yinhuapinggan granule (YHPG)

YHPG is optimized from the classic ephedra decoction and can alleviate cough. It consists of the root of *P. l*obata (Willd.) Ohwi, *L. japonica* Thunb., *Reynoutria japonica* Houtt., *E. sinica* Stapf, *P. armeniaca*, and *G. glabra* L. (Jin et al., 2022). YHPG inhibited the proliferation of PR8 in MDCK cells. In addition, in PR8-infected ICR mice, YHPG alleviated the lung damage, decreased the expression levels of IL-4, IL-5, and TNF-α but upregulated the levels of IL-2 and IFN-γ in serum, and significantly downregulated the mRNA expression levels of TLR4, MyD88, TRAF6, and NF-κB p65 in the lungs. It is proposed that the potential mechanism of YHPG in treatment of influenza pneumonia is related to the inhibition of cytokine storm mediated by the TLR4/NF-κB signaling pathway (Peng et al., 2016a; Peng et al., 2016b).

#### 3.1.9 Luofushan-baicao oil (LBO)

LBO is an essential oil-rich TCM formula and usually used to alleviate cold, headache, insect bites, and swelling (Hayashi et al., 2007; Wu et al., 2010; Li Y. et al., 2016; Mao et al., 2021). It consists of seventy-nine kinds of botanical drugs and related extractions, and the detailed composition of this formula was reported in the study by Mao et al. (Mao et al., 2021). LBO significantly inhibited the proliferation of influenza virus A/WSN/1933 (H1N1) (WSN) and virus titer in MDCK cells and reduced the expression of viral nuclear protein, IL-1β, IL-6. In a mouse model infected with FM1, inhalation of LBO aerosol reduced the lung damage and the lung index, and decreased the serum IL-1β and IL-6 levels. Further, in Poly I: C-stimulated A549 cells, the mRNA expression of IL-1β, IL-6, and IFN-β as well as the protein expression of NF-κB p65 and IRF3 were significantly lower in LBO treatment group. LBO has been suggested as an inhibitor of the TLR3/NF-κB signaling pathway to prevent influenza virus infection and balance immune function that it could be developed as a substitute medicinal agent for the prevention of influenza (Mao et al., 2021).

#### 3.1.10 KangBingDu (KBD) oral liquid

KBD is a Chinese patent drug developed from the ‘Baihu Decoction’ and ‘Qingwen Baidu Yin’ and is widely used for treatment of viral infections (Li et al., 2022). It consists of *I. tinctoria* L., *P. australis*, *Rehmannia glutinosa* (Gaertn.) DC., *Curcuma aromatica* Salisb., *Anemarrhena asphodeloides* Bunge, *Acorus calamus*, *Pogostemon cablin* (Blanco) Benth., *F. suspensa* (Thunb.) Vahl, and gypsum fibrosum. It has been demonstrated that KBD inhibited viral loads, increased survival rate, and attenuated lung damage in FM1-infected Kunming mice. KBD treatment upregulated the protein levels of MAVS, phospho-IRF3, and downstream IFN-β. Meanwhile, the suppression of protein levels of NF-κB p65, TNF-α, and IL-1β were observed, whereas viral infection induced the expression of these proteins. It was proposed that KBD can alleviate lung injury and exert anti-influenza effect through the impact on the activation of the MAVS/NF-κB signaling pathway (Chen et al., 2017).

### 3.2 The single-active-ingredients of TCMs

#### 3.2.1 Carvacrol

Carvacrol is one of the main components of *M. chinensis Maxim*, which has analgesic, anti-inflammatory, antiviral, insecticidal, and antioxidant effects (Suntres et al., 2015). It was demonstrated that carvacrol significantly reduced the lung index and the expression levels of IFN-γ, IL-2, IL-4, IL-5, and IL-10 in the lung tissue of FM1-infected C57BL/6 mice. Furthermore, following the treatment with carvacrol, the mRNA expression levels of TLR7, MyD88, IRAK4, NF-κB p65, MAVS, and IRF3 were decreased, and the protein expression levels of RIG-I, MyD88, and NF-κB were significantly downregulated. It has been suggested that carvacrol may regulate excessive secretion of inflammatory factors *via* inhibiting both TLR and RIG-I signaling pathways which were activated under influenza virus infection so as to reduce lung damage resulting from viral infection (Zheng et al., 2021).

#### 3.2.2 C8

The compound C8 was isolated from the botanical drug choulingdan (*Laggera crispata*), which is often used to treat diseases such as pharyngitis and stomatitis (Wu et al., 2006; Shi et al., 2007; Wang et al., 2017). The main content of C8 includes 107.98 mg/g odontodanic acid and 111.3 mg/g odontodandiol. C8 showed broad-spectrum anti-influenza activity that can inhibit replication of H1N1, H6N2, H7N3, and H7N9 influenza viruses in MDCK cells (Guan et al., 2017; Wang et al., 2017). It significantly inhibited the mRNA expression levels of IL-1β, IL-6, IL-8, and MCP-1, as well as the protein levels of TLR7, MyD88, and TRAF6 and the nuclear translocation of NF-κB p65 in PR8-infected A549 cells. C8 is expected to target the TLR7/NF-κB signaling pathway to interfere with the infection of influenza virus (Wang Y. et al., 2018).

#### 3.2.3 Berberine

Berberine is an isoquinoline alkaloid isolated from a Chinese botanical drug huanglian (*C. chinensis* Franch.) and has anti-hypertensive (Guo et al., 2015), anti-inflammatory (Liu S. J. et al., 2015) and antiviral effects (Kuo et al., 2004; Shin et al., 2015). The anti-influenza activity of berberine was studied using A549 cells infected with FM1 virus, and the results showed that this drug inhibited viral proliferation in a time- and dose-dependent manner. In C57BL/6 mice, viral infection induced tissue damage and the expression of IL-4, TNF-α, and IFN-γ in the lungs, which were alleviated following the treatment of berberine. Moreover, the mRNA and protein expression levels of TLR7, MyD88, and NF-κB p65 in the lungs of mice were significantly increased after viral infection but significantly downregulated in berberine treatment group. However, further investigations are needed to see whether berberine has a direct effect on the above signaling pathways (Wu Y. et al., 2011; Yan et al., 2018).

#### 3.2.4 Forsythiaside A

Lianqiao (*F. suspensa* (Thunb.) Vahl) is a well-known Chinese botanical drug and plays an important role in treating fever, inflammation, ulcers, and gonorrhea (Qu et al., 2008). So far, over 300 ingredients have been extracted from lianqiao (*F. suspensa* (Thunb.) Vahl). Among them, the main bioactive index component is forsythiaside A, which has anti-bacterial, antioxidant, and antiviral activities (Deng et al., 2016; Zeng et al., 2017; Zhou et al., 2022). In FM1-infected C57BL/6 mice, forsythiaside A reversed weight loss and alleviated lung damage and significantly reduced the mRNA and protein expression levels of TLR7, MyD88, and NF-κB p65 in the lung tissues. However, the mRNA and protein expression levels of MyD88 and NF-κB p65 in the lung tissues of TLR7-deficient mice infected with FM1 were similar between the forsythiaside A treatment group and the viral infection group. Forsythiaside A was indicated to interfere with the activation of the TLR7 signaling pathway to influence the inflammatory response and the subsequent lung injury caused by viral infection (Zheng et al., 2022).

#### 3.2.5 Polysaccharides isolated from *Houttuynia cordata* thunb (HCP)

Yuxingcao (*H. cordata* Thunb.) is an important botanical drug with antiviral, antibacterial, anti-inflammatory, and antioxidant activities (Li et al., 2013; Cheng et al., 2014; Ling et al., 2023). HCP, one of the active components of this botanical drug, alleviated pulmonary and intestinal damages in BALB/c mice infected with FM1, and improved the survival rate of the infected mice. Moreover, HCP not only inhibited excessive release of TNF-α, IL-6, and IFN-α but also suppressed the upregulation of TLR4 and NF-κB protein expression in the lung tissues of infected mice. It is possible that HCP acts against influenza virus infection by inhibiting the activation of the TLR4/NF-κB signaling pathway (Zhu et al., 2018).

#### 3.2.6 Ergosterol peroxide

Ergosterol peroxide isolated from the root of nanbanlangen (*Strobilanthes cusia* (Nees) Kuntze) has been proved to have anti-tumor, antioxidant, and antibacterial effects (Ramos-Ligonio et al., 2012; Wu et al., 2012; Kang et al., 2015). It was demonstrated that in A549 cells infected with PR8 strain, ergosterol peroxide inhibited the overexpression of pro-inflammatory cytokines, including TNF-α, IL-6, IL-8, IP-10, CCL5, MCP-1, and MIP-1α. Meanwhile, this drug significantly downregulated the mRNA and protein expression levels of RIG-I in PR8-infected cells. The transcriptional activity of NF-κB stimulated by PR8 virus or by TNF-α treatment was significantly lower following ergosterol peroxide treatment. It has been speculated that the anti-inflammation activity of ergosterol peroxide from banlangen under influenza virus infection is mediated *via* inhibition of the obstructing RIG-I signaling pathway (Zhou et al., 2019).

#### 3.2.7 Baicalin

Baicalin is a flavonoid isolated from the root of a botanical drug huangqin (*S. baicalensis* Georgi), and it has been used for treating cough (Ding et al., 2014; Zhu et al., 2016). It was demonstrated that baicalin reduced the lung index, and pulmonary damage, and the secretion of IFN-γ, IL-1β, IL-2, IL-5, and IL-6 in the lung tissues of FM1-infected C57BL/6 mice. Meanwhile, the transcriptional and translational levels of RIG-I, IRF3, IRF7, and NF-κB in the lung tissues were significantly increased in response to viral infection, while the treatment of baicalin significantly decreased the expression and production of these genes. The effects of baicalin on several factors in RIG-I signaling pathway highlighted the advantages of TCM in regulating host immunity (Pang et al., 2018).

#### 3.2.8 Rhein

Rhein is extensively found in several TCMs, including *Rheum palmatum* L., *Aloe barbadensis* Miller, *Cassia angustifolia* Vahl., and *Polygonum multiflorum* Thunb. It has antioxidant, antiviral, anti-inflammatory, anti-tumor, and immunomodulatory activities (Sun et al., 2016). A recent study has shown that rhein significantly inhibited the proliferation of influenza virus A/ShanTou/169/06 (H1N1) (ST169) in MDCK cells and reduced the expression levels of IL-1β, IL-6, IL-8, and TNF-α in PR8-infected A549 cells. In mice infected with PR8, rhein significantly improved the survival rate and reduced the lung index and pulmonary expression levels of IL-1β, IL-6, IL-8, TNF-α. Further, rhein significantly reduced the protein levels of TLR2, TLR3, and TLR4, as well as the phosphorylation of NF-κB p65 in PR8-infected A549 cells. The addition of activators for TLR4 and NF-κB could antagonize the inhibitory effect of rhein on virus replication (Wang Q. W. et al., 2018). Thus, the anti-influenza effect of rhein is likely related to inhibiting the activation of TLRs/NF-κB signaling pathway which may also alleviate the production of pro-inflammatory cytokines.

#### 3.2.9 Polyphenol extract from the bark of houpu (MPE)

MPE is a polyphenol compound extracted from the bark of houpu (the bark of *M. officinalis*) (Sarrica et al., 2018). It was reported that MPE reduced the mortality rate and the lung index, improved inflammatory changes of lung tissues, and decreased the serum levels of TNF-α and IL-6 in a dose-dependent manner in PR8-infected ICR mice. Further, MPE significantly reduced the protein level of TLR3 and phospho-NF-κB p65 in the lung tissues. It is proposed that MPE may play a role in regulating the immune response and alleviating viral pneumonia by inhibition of the activation of TLR3/NF-κB signaling pathway (Wu X. N. et al., 2011).

#### 3.2.10 Patchouli alcohol

Patchouli alcohol is the main component of guanghuoxiang (*P. cablin* (Blanco) Benth.) which is a botanical drug (Kiyohara et al., 2012). Following FM1 virus infection, patchouli alcohol inhibited the expression of pro-inflammatory cytokines such as IFN-γ and IL-4 in HBE cells, and significantly downregulated the mRNA expression levels of RIG-I, MAVS, IRF3, and IRF7 in dendritic cells and macrophages (Wu et al., 2013). It has been suggested that patchouli alcohol plays antiviral and anti-inflammatory roles *via* regulating the innate immune response. However, the detailed effects on the virus and these immune factors are needed to verify this speculation.

#### 3.2.11 (2Z,4E)-deca-2,4-dienoic acid (DDEA)

Wild dongchongxiacao (cordyceps) mainly consists of the stroma of the fungus *Cordyceps sinensis* (Berk.) Sacc. and the dead caterpillar of *Hepialus armoricanus*, which is a rare TCM with immunomodulatory activity (Ng and Wang, 2005; Paterson, 2008; Liu Y. et al., 2015). DDEA was a novel fatty acid identified in *Ophiocordyceps sinensis* which has been cultured as an alternative of the wild dongchongxiacao. In A549 cells infected with PR8, the chemically synthesized DDEA could not inhibit viral proliferation but inhibited the mRNA and protein expression of TNF-α, IFN-α, IFN-β, IL-6, IL-8, MCP-1, IP-10, and MIP-1β. Moreover, DDEA significantly reduced the protein expression levels of TLR3, RIG-I, phospho-IRF3, and phospho-NF-κB p65 in infected cells. It is suggested that DDEA may regulate the immune function by regulating TLR3 and RIG-I-dependent signaling pathways (Li et al., 2021).

## 4 Conclusion

TCMs are derived from natural plants and have advantages such as wide clinical applications and negligible side effects. Here we reviewed the research progress of the mechanism of different sorts of TCMs against influenza, including prescriptions, the Chinese patent drugs, and active ingredient. A variety of TCMs can inhibit the replication of influenza viruses and improve the symptoms of infected mice. However, for many TCMs, it is still unclear on which stage in the viral replication cycle they act. The investigation of the mode of action of TCMs would provide hints for their clinical use. Besides the antiviral effects, TCMs exhibit immune modulatory effects including regulating the expression of PRRs such as TLR3, TLR4, TLR7, RIG-I, and MAVS, and the key proteins of the NF-κB signaling pathway and the inhibition of the pro-inflammatory response. Interestingly, TLR7/MyD88/NF-κB signaling pathway was more frequently reported to be interfered by several formulae and active ingredients of TCMs. Although several studies demonstrated that the virus replication triggers the activation of inflammatory responses, whether the decrease in PRR expression and NF-κB activation is secondary to a decrease in viral burden was less analyzed. In addition, it has not yet been conclusively demonstrated whether the drugs inhibit the pro-inflammatory response by affecting key proteins of these signaling pathways and thus inhibit the occurrence of viral pneumonia. Some of the proinflammatory factors directly induced by virus infection could further induce a second wave of cytokines, which complexes the immune responses induced by viral infection. Recent studies have begun to recognize the multifaceted roles of cytokines during influenza infection, but the function and place of immune effectors in the immune network are still needed to be defined (Guo and Thomas, 2017). To provide support for research of anti-influenza drugs, the relationship between virus and signaling elements should be further investigated. The current studies mainly focus on the effects of TCMs against influenza A virus, while the activity of these drugs against influenza B virus is less reported. It should be noted that the immunomodulatory role of drugs has a wide array of potential uses and TCMs have the advantage of providing complementary treatment for new emerging diseases. Therefore, TCM targeting PRRs might also yield some benefit to other illnesses related to host immunopathology and acute lung injury beyond Influenza. In a word, the detailed roles of PRR-mediated NF-κB signaling pathways in regulating the inflammatory responses and the efficient pharmacodynamic substances should be further investigated while retaining the characteristics of TCMs, which will help promote global recognition of TCMs.
